# Morphologische und funktionelle Folgen nach COVID-19-Pneumonie

**DOI:** 10.1007/s00117-021-00905-4

**Published:** 2021-09-16

**Authors:** Ruxandra-Iulia Milos, Daria Kifjak, Benedikt H. Heidinger, Florian Prayer, Lucian Beer, Sebastian Röhrich, Christian Wassipaul, Daniela Gompelmann, Helmut Prosch

**Affiliations:** 1 Universitätsklinik für Radiologie und Nuklearmedizin, Medizinische Universität Wien, Währinger Gürtel 18–20, 1090 Wien, Österreich; 2 Klinische Abteilung für Pulmologie, Universitätsklinik für Innere Medizin II, Medizinische Universität Wien, Wien, Österreich; 3 Universitätsklinik für Thoraxchirurgie, Medizinische Universität Wien, Wien, Österreich

**Keywords:** Long-COVID, Post-COVID-Klinik, Diagnostische Bildgebung, Lungenfunktion, Nachsorge, Long COVID, Post-COVID clinics, Diagnostic imaging, Pulmonary function, Follow-up

## Abstract

**Hintergrund:**

Nach einer Coronavirus-Krankheit-2019 (COVID-19) berichtet ein Teil der Patienten über länger andauernde oder sich verschlechternde Symptome und Beeinträchtigungen. Diese anhaltenden Symptome werden mit dem Begriff „Long-COVID“-Syndrom zusammengefasst. Sie können mit radiologischen Veränderungen in der Computertomographie (CT) und einer Verschlechterung der Lungenfunktion einhergehen.

**Ziel der Arbeit:**

Die Rolle der langfristigen Verlaufskontrollen von COVID-19-Patienten wird erörtert, um festzustellen, welche Patienten davon profitieren können.

**Material und Methoden:**

In diesem Artikel werden die aktuellen Ergebnisse der klinischen, radiologischen und lungenfunktionellen Nachsorgenuntersuchungen nach COVID-19-Pneumonie präsentiert.

**Ergebnisse:**

Chronische Müdigkeit und Dyspnoe sind die häufigsten anhaltenden Symptome nach COVID-19. Außerdem zeigen viele dieser Patienten eine Beeinträchtigung der körperlichen Leistungsfähigkeit. In der CT sind Milchglasareale und strangförmige Verdichtungen die häufigsten residualen Veränderungen nach einer COVID-19-Pneumonie, die histologisch einer organisierenden Pneumonie entsprechen. Ein Teil der Patienten kann nach einer schweren COVID-19-Pneumonie im Verlauf *fibroseähnliche* Veränderungen aufweisen. Patienten mit einem vormals schwereren Verlauf können ein restriktives Syndrom mit niedriger Kohlenmonoxid-Diffusionskapazität (DLCO) und Gesamt-Lungenkapazität (TLC) zeigen. Im längerfristigen Verlauf zeigen die meisten Patienten eine deutliche und kontinuierliche Verbesserung aller Symptome sowie einen Rückgang der radiologisch-morphologischen und funktionellen Veränderungen.

**Diskussion:**

Patienten mit persistierenden Symptomen nach COVID-19 sollten in spezialisierten Post-COVID-19-Ambulanzen multidisziplinär abgeklärt und behandelt werden.

## Abstract (en)

**Background:**

Following coronavirus disease 2019 (COVID-19), a proportion of patients report prolonged or worsening symptoms and impairments. These symptoms are increasingly referred to as “long COVID” syndrome. They may be associated with radiological changes on computed tomography (CT) and pulmonary function impairment.

**Objectives:**

To discuss the role of long-term assessment of COVID-19 patients to determine which patients may benefit from follow-up.

**Materials and methods:**

This article presents the current results of clinical, radiological, and pulmonary function follow-up tests after COVID-19 pneumonia.

**Results:**

Chronic fatigue and dyspnea are the most common persistent symptoms after COVID-19. Patients also present impaired exercise capacity. On CT, ground-glass opacities and parenchymal bands are the most common residual changes after COVID-19 pneumonia, histologically corresponding to organizing pneumonia. A proportion of patients who had severe COVID-19 pneumonia may show *fibrotic-like* changes during follow-up. Patients with severe acute infection may present with a restrictive syndrome with lower diffusing capacity for carbon monoxide (DLCO) and total lung capacity (TLC) values. Overall, significant and continuous improvement in all symptoms as well as radiomorphological and functional changes were observed over time.

**Conclusions:**

Patients with persistent symptoms after COVID-19 should be evaluated and treated in specialized post-COVID-19 clinics in a multidisciplinary manner.

## Hintergrund

Die Coronavirus-Krankheit-2019 („coronavirus disease 2019“, COVID-19) verursacht eine Vielfalt an Symptomen, die von einer leichten asymptomatischen Erkrankung bis hin zu einem schweren respiratorischen Versagen reichen. Während bei den meisten Patienten die Symptome der Erkrankung innerhalb von 4 Wochen vollständig abklingen, berichtet ein Teil der Patienten über länger andauernde Symptome und Beeinträchtigungen. Dazu gehören pulmonale Symptome wie chronischer Husten, Belastungsdyspnoe sowie ein thorakales Engegefühl [33]. Zusätzlich finden sich auch extrapulmonale Symptome wie Anosmie, Anorexie mit Gewichtsverlust, Parästhesien, kognitive Dysfunktion und Müdigkeit [33]. Diese Symptome werden zunehmend als „Long-COVID“-Syndrom bezeichnet. Abhängig von der Dauer der Symptome und den assoziierten Organmanifestationen wurden mehrere Phasen der Coronavirus-Infektion definiert. Symptome der Erkrankung, die über die akute Phase von 4 Wochen hinausgehen, werden allgemein als Long-COVID bezeichnet [12, 36]. Manche Autoren unterteilen zusätzlich das Long-COVID-Syndrom in ein „anhaltend symptomatisches COVID-19“, wenn die Symptome 4 bis 12 Wochen nach Erkrankungsbeginn bestehen, und in ein „Post-COVID-19-Syndrom“, wenn die Symptome über 12 Wochen nach Erkrankungsbeginn hinaus andauern ([12, 36], Tab. 1, mod. nach [12]).

**Table 1:** 

Akutes COVID-19	COVID-19-Veränderungen und Symptome für bis zu 4 Wochen
Anhaltend symptomatisches COVID-19	COVID-19-Veränderungen und Symptome nach 4 bis zu 12 Wochen
Post-COVID-19-Syndrom	COVID-19-Veränderungen und Symptome, die sich während oder nach einer COVID-19-Infektion entwickeln, länger als 12 Wochen andauern und nicht durch eine alternative Diagnose erklärt werden können
Long-COVID-19	COVID-19-Veränderungen und Symptome, die nach einer akuten COVID-19 fortbestehen oder sich entwickeln; dies umfasst sowohl das anhaltend symptomatische COVID-19- (nach 4 bis 12 Wochen) als auch das Post-COVID-19-Syndrom (nach ≥ 12 Wochen)

Während die genauen Mechanismen für das Fortbestehen der Symptome derzeit nicht bekannt sind, reichen die Hypothesen diesbezüglich von virusspezifischen pathophysiologischen Veränderungen, wie z. B. die Invasion von Alveolarepithel- und Endothelzellen durch das Virus, immunologischen Phänomenen und entzündlichen Schäden als Reaktion auf die akute Infektion bis hin zu einem „Post-Intensive-Care“-Syndrom (ICU-Syndrom; [24]). Eine wichtige Rolle in der Pathogenese von COVID-19 spielen diffuse vaskuläre Schäden durch Endotheliitis, Thrombose und Angiogenese [1]. Eine dadurch verursachte anhaltende vaskuläre Dysfunktion könnte auch eine Rolle bei der postakuten Symptomatik und Organdysfunktion spielen.

Das Risiko, ein Long COVID-Syndrom zu entwickeln, scheint von mehreren Faktoren abhängig zu sein, wobei in der aktuellen Literatur kontroverse Aussagen zu finden sind. Als Risikofaktoren werden der Schweregrad der akuten Erkrankung, höheres Alter, weibliches Geschlecht und ein höherer Body-Mass-Index (BMI) diskutiert [27, 32].

## Klinische Manifestationen nach pulmonaler COVID-19-Infektion

Im Zusammenhang mit COVID-19 wurde von Patienten sowohl über anhaltende Symptome als auch über neue oder sich verschlechternde Symptome berichtet. Derzeit ist allerdings nicht klar, ob Long-COVID entweder eine Erweiterung eines akuten Post-COVID ist oder einen separaten Krankheitssubtyp darstellt, welcher ein anderes Risikoprofil aufweist.

### Klinische Merkmale des anhaltenden symptomatischen COVID-19-Syndroms

Chronische Müdigkeit und Dyspnoe sind die häufigsten persistierenden Symptome nach akutem COVID-19, mit einer gepoolten Prävalenz von 52 und 37 % [4]. Thoraxschmerzen und Husten zeigten in einer Metaanalyse eine gepoolte Prävalenz zwischen 14 und 16 % [4]. Des Weiteren waren die Ergebnisse des 6‑Minuten-Gehtests bei den COVID-19-Patienten in der frühen Rekonvaleszenzphase im Vergleich zu den Referenzwerten signifikant reduziert [14, 19]. Zudem benötigten ca. 6 % der Patienten eine Sauerstofftherapie oder eine Atemunterstützung während des Schlafs aufgrund einer anhaltenden Hypoxämie [5]. In einer Studie mit über 1800 Patienten, die während akutem COVID-19 eine Tracheostomie benötigten, konnten nur 52 % der Patienten einen Monat später erfolgreich von der mechanischen Beatmung entwöhnt werden; 24 % der Patienten wurden weiterhin mechanisch beatmet [6]. Sechs Wochen nach der Entlassung litten hospitalisierte Patienten mit schwerem COVID-19 unter einer verminderten Lebensqualität, hauptsächlich aufgrund einer eingeschränkten Mobilität [7].

### Klinische Merkmale des Post-COVID-19-Syndroms

Sechs Monate nach der akuten Infektion gaben 76 % der hospitalisierten Patienten mindestens ein Symptom an, wobei Frauen häufiger betroffen waren als Männer [18]. Dyspnoe und Brustschmerzen wurden von 26 % bzw. von 5 % der Patienten berichtet [18]. Außerdem berichteten ca. 50 % der zu Hause isolierten jungen Erwachsenen im Alter von 16–30 Jahren 6 Monate nach der Infektion über Symptome wie Müdigkeit (21 %) und Dyspnoe (13 %; [3]). Bei Patienten nach einem COVID-19-ARDS („acute respiratory distress syndrome“, Atemnotsyndrom des Erwachsenen) waren die häufigsten Symptome nach 6 Monaten Müdigkeit (44 %) und Abgeschlagenheit (33 %), während respiratorische Symptome wie Dyspnoe und Husten weniger häufig berichtet wurden (18 und 17 %; [8]). Der 6‑Minuten-Gehtest lag in dieser Studie nur bei 2 Patienten (11 %) unter der unteren Normgrenze [8]. Bei keinem Patienten gab es einen signifikanten Abfall der Sauerstoffsättigung nach Belastung [8]. Insgesamt wurde mit der Zeit eine deutliche und kontinuierliche Verbesserung aller Symptome (Beeinträchtigung der körperlichen Leistungsfähigkeit, Dyspnoe, Husten usw.) beobachtet [34].

## CT-Veränderungen bei Long-COVID

Bei Patienten mit Long-COVID-Syndrom können pulmonale Symptome mit radiologischen Veränderungen und Lungenfunktionsbeeinträchtigungen vergesellschaftet sein. Allerdings können auch asymptomatische Patienten morphologische Veränderungen des Lungenparenchyms in der Computertomographie (CT) aufweisen oder symptomatische Patienten eine unauffällige CT zeigen.

### CT-Veränderungen der Lunge bei akuter COVID-19-Pneumonie und anhaltendem symptomatischem COVID-19-Syndrom

Die CT-Veränderungen des Lungenparenchyms im Rahmen einer COVID-19-Pneumonie zeigen eine zeitliche Dynamik. Die ersten Veränderungen sind meist Milchglasverdichtungen mit oder ohne Konsolidierungsarealen sowie verdickten Interlobärsepten in Kombination mit Milchglas („crazy paving“; [17, 25]). Im weiteren Verlauf kann es zur Bildung von strangförmigen, arkadenartigen, subpleuralen Verdichtungen kommen, die als „fibrous stripes“ bezeichnet werden [26] und histologisch einer organisierenden Pneumonie entsprechen [28].

Diese Veränderungen nehmen im Verlauf der Erkrankung an Dichte und Ausdehnung ab. Nach dem Abklingen der akuten Phase kommt es 3 bis 4 Wochen nach der Entlassung bei 53–65 % der Patienten zu einer vollständigen Rückbildung der Lungenparenchymveränderungen [20, 21]. Patienten unter 44 Jahren zeigten 3 Wochen nach Entlassung signifikant häufiger eine vollständige radiologische Rückbildung als Patienten über 44 Jahren [21].

**Figure 1:**
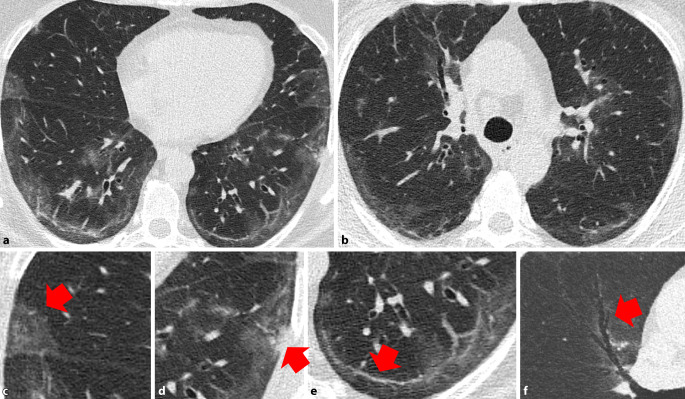

**Figure 2:**
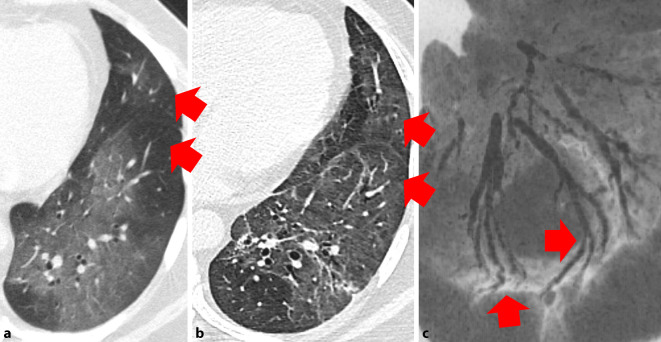

Die am häufigsten beobachteten residualen Veränderungen des Lungenparenchyms sind Milchglasverdichtungen, gefolgt von strangförmigen, subpleuralen Verdichtungen und Verdickung der angrenzenden Pleura (Abb. 1, [20, 21]). Interessanterweise wurden zusätzlich zwei besondere Merkmale beobachtet:

1. bronchovaskuläre Distorsion (Abb. 2),
2. eine vorübergehende, progrediente Ausdehnung der Milchglasverdichtungen mit begleitender Dichteabnahme, ein Phänomen, das als „tinted sign“ bezeichnet wurde (Abb. 2) und möglicherweise eine graduelle Rückbildung der Entzündung assoziiert mit Reexpansion der Alveolen widerspiegelt [21].

Alle CT-morphologischen Veränderungen zeigten im Verlauf eine graduelle Regredienz [20].

### CT-Veränderungen der Lunge bei Post-COVID-19-Syndrom

**Figure 3:**
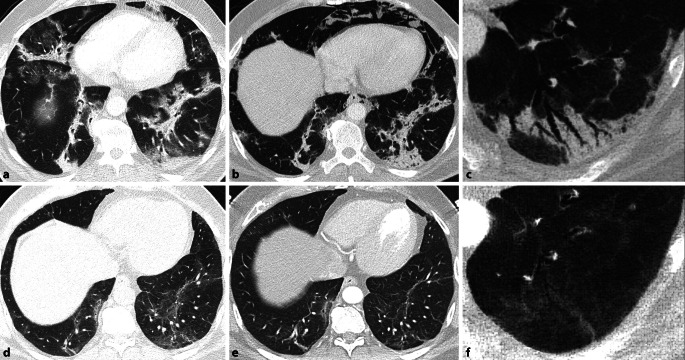

**Figure 4:**
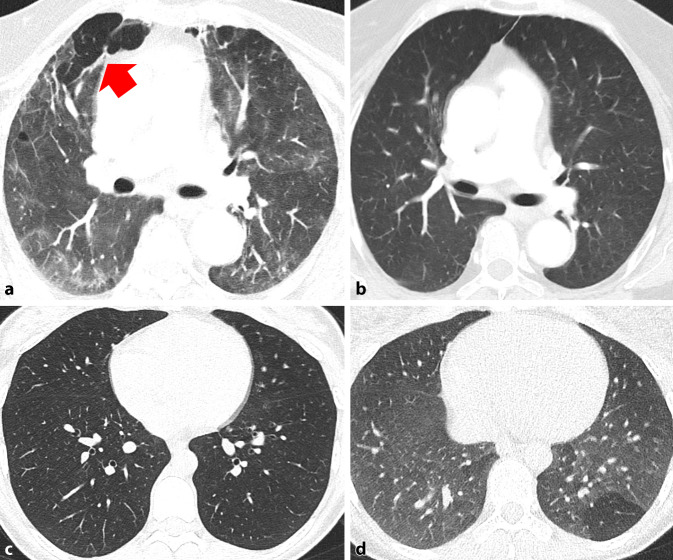

Eine Metaanalyse von 15 Studien über 3066 Patienten mit unterschiedlich schweren Erkrankungsformen konnte zeigen, dass bei einer Nachbeobachtungszeit von 1–6 Monaten die häufigsten beschriebenen CT-Auffälligkeiten Milchglasveränderungen (in 44 %), gefolgt von strangförmigen Verdichtungen (34 %) waren ([30], Abb. 3). In einem Teil der Patienten wurde 3 bis 4 Monate nach der Entlassung ein Mosaikmuster beobachtet, welches entweder auf eine residuale Erkrankung der kleinen Atemwege oder auf eine mikrovaskuläre Thrombose zurückzuführen sein könnte ([9, 15], Abb. 4). Dieses Mosaikmuster entspricht „air trapping“, welches auch auf exspiratorischen Scans bestätigt werden konnte (Abb. 4).

Aufgrund des Ausmaßes der Lungenbeteiligung, begleitenden Pathologien und der mechanischen Beatmung bilden die COVID-19-ARDS-Überlebenden eine besondere Gruppe von Patienten. Drei Monate nach COVID-19 zeigten die CT-Scans bei 70–80 % dieser Patienten weiterhin Auffälligkeiten. Insgesamt wiesen diese Patienten ein breites Spektrum an Veränderungen wie Milchglas und retikulären Verdichtungen allein oder in Kombination, Konsolidierungen, interlobulären septalen Verdichtungen, Bronchiektasien und Architekturstörungen auf ([2, 11, 14], Abb. 3). Das Ausmaß der persistierenden radiologischen Läsionen korrelierte mit der Anzahl der Tage auf der Intensivstation, während die mechanische Beatmung mit Zeichen, die auf eine Fibrose hinweisen (Kombination von Retikulationen und Traktionsbronchiektasien) assoziiert war [11]. Der Begriff „Traktionsbronchiektasie“, der auf irreversible fibrotische Veränderungen hindeutet, sollte in diesem Zusammenhang jedoch mit Vorsicht verwendet werden, da eine bronchiale Dilatation und Distorsion in Bereichen von Konsolidierungen oder Milchglasverdichtungen im längerfristigen Verlauf reversibel sein kann (Abb. 3, [22]). Daher sollten diese Veränderungen als „fibroseähnliche Veränderungen“ bezeichnet werden, die wahrscheinlich Residuen einer (abgelaufenen) organisierenden Pneumonie widerspiegeln [22]. Ein Honeycombing, welches das definitive CT-Merkmal einer Lungenfibrose darstellt, wurde nur in anekdotischen Fallberichten beschrieben. Dieses kann möglicherweise mit einer vorbestehenden fibrosierenden Lungenerkrankung in Verbindung gebracht werden [22].

Bei den meisten Patienten wurde im Verlauf eine radiologische Verbesserung beobachtet (Abb. 3). Allerdings zeigten in der CT 35 % der Überlebenden einer schweren COVID-19-Pneumonie noch 6 Monate nach COVID-19-Pneumonie fibroseähnliche Veränderungen in der CT. Die verbleibenden 65 % dieser Patienten zeigten entweder eine vollständige radiologische Rückbildung (38 %) oder residuelle Milchglasverdichtungen und interlobuläre septale Verdichtungen (27 %; [16], Abb. 3).

Bei 24 % der Patienten mit einer schweren COVID-19-Pneumonie, jedoch ohne invasiver Beatmung, fanden sich 12 Monate nach Entlassung immer noch persistierende radiologische Auffälligkeiten, überwiegend Milchglasverdichtungen [34]. Bei diesen Patienten wurde nach 12 Monate keine signifikante radiologische Verbesserung im Vergleich zu 9 Monaten nach Entlassung festgestellt (Abb. 3), und keiner der CT-Scans zeigte Hinweise auf eine definitive Lungenfibrose oder auf progressive interstitielle Veränderungen ([34], Abb. 3).

**Figure 5:**
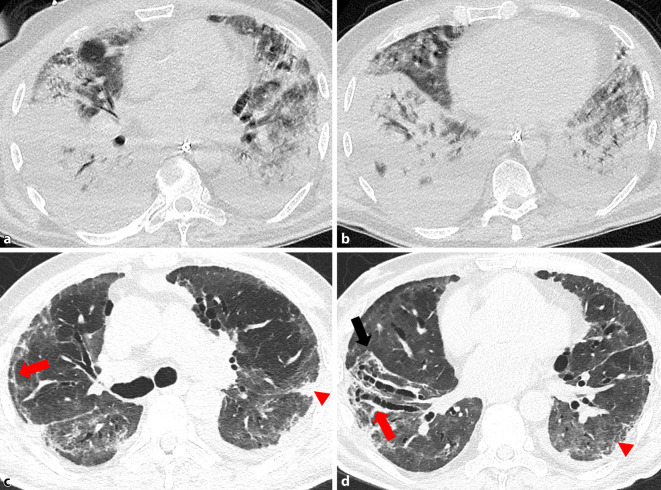

Andererseits können Überlebende einer schweren COVID-19-Pneumonie mit invasiver Beatmung in der Spätphase zunehmende Zeichen einer Parenchymdestruktion mit Fibrose entwickeln (Abb. 5). Diese sind allerdings in erster Linie auf Folgen von Alveolarschäden im Rahmen des ARDS zurückzuführen und daher weniger charakteristisch für COVID-19.

Neben den oben beschriebenen pulmonalen Veränderungen ist eine COVID-19-Erkrankung auch ein Risikofaktor für die Entwicklung thrombotischer und embolischer Ereignisse. Diese Ereignisse können ebenfalls mit längerfristigen Veränderungen assoziiert sein. Die Dual-Energy-CT (DECT) ermöglicht die Beurteilung der pulmonalen Perfusion und die Erkennung kapillarer mikrovaskulärer Thrombosen [1]. Die DECT-Angiographie 3 Monate nach COVID-19-Pneumonie zeigte in einer Studie bei 5,4 % der Patienten eine proximale arterielle Thrombose und bei 65,5 % der Patienten Perfusionsanomalien, die auf eine weit verbreitete Mikroangiopathie hinwiesen. Vier Patienten mit normalem Lungenparenchym zeigten eine gestörte Mikrozirkulation der Lunge [29].

## Funktionelle Folge der COVID-19-Pneumonie und Korrelation mit CT-Veränderungen

Mehrere Studien untersuchten die Lungenfunktion (LUFU) sowie die Diffusionskapazität zu verschiedenen Zeitpunkten nach einer COVID-19-Pneumonie: bei Entlassung [23], nach 30 Tagen [19], nach 3 bis 4 Monaten [10, 15, 35], nach 6 Monaten [18, 31] und nach 12 Monaten [34]. Insgesamt war die Diffusionskapazität (DLCO) einheitlich reduziert, während die forcierte Vitalkapazität (FVC) und das forcierte exspiratorische Volumen in 1 s (FEV_1_) weniger betroffen waren. Patienten mit einer schwereren akuten Infektion hatten niedrigere DLCO- und Gesamtlungenkapazitätswerte (TLC; [15, 18, 19, 23]). Ein höherer D‑Dimer-Wert bei Aufnahme konnte eine beeinträchtigte DLCO 3 Monate nach Entlassung vorhersagen [35], möglicherweise sekundär in Zuge mikrovaskulärer Thrombosen. Huang et al. beobachteten, dass ungefähr die Hälfte der Patienten, welche mit einer mechanischen Beatmung behandelt wurden, nach 6 Monaten weiterhin einen DLCO-Wert unter 80 % aufwiesen [18]. Zudem konnte gezeigt werden, dass eine restriktive Ventilationsstörung (Verminderung der TLC) mit einer vorangegangenen Intubation, neuromuskulären Blockade und der sog. Critical-illness-Polyneuropathie assoziiert war [31]. Die Restriktion verbesserte sich sukzessiv im Laufe der Zeit, war aber nach 12 Monaten nicht vollständig behoben [34]. Obstruktive Muster bei der Spirometrie wurden in diesen Studien selten beobachtet. Die arterielle Blutgasanalyse wurde nicht systematisch in den bereits publizierten Studien beschrieben.

Eine eindeutige Korrelation zwischen der niedrigen DLCO bei Patienten mit Long-COVID und den residualen Veränderungen in der Thorax-CT konnte bisher nicht nachgewiesen werden. In der frühen Rekonvaleszenzphase wurde keine signifikante Korrelation zwischen den Lungenfunktionsparametern und dem Schweregrad der Lungenveränderungen in der CT gefunden [19]. Im Gegensatz dazu wurde in der Studie von Frija-Masson et al. eine signifikant niedrigere Lungenfunktion bei Patienten mit residualen CT-Läsionen 3 Monate nach der COVID-19-Pneumonie berichtet [10]. In einer längerfristigen Beobachtungszeit von 6 Monaten verbesserten sich die klinischen Symptome und der Thorax-CT-Score unabhängig von der Restriktion [31].

## Empfehlungen für Post-COVID-19-Nachsorge

Zurzeit gibt es nur wenige, spezifische Richtlinien für die langfristige Nachsorge von Patienten nach einer COVID-19-Pneumonie [12, 13]. Die Schweizer COVID-Lungenstudiengruppe und die Schweizer Gesellschaft für Pulmologie empfehlen ein 3‑monatiges pulmonales Follow-up für:

1. alle Patienten, die entweder hospitalisiert waren und/oder einen schwereren klinischen Verlauf der Erkrankung aufwiesen,
2. alle symptomatischen Patienten, auch nach mildem Erkrankungsverlauf. Bei allen symptomatischen Patienten wird die Durchführung einer Thorax-CT Untersuchung 3 Monate nach Krankenhausentlassung empfohlen.

In allen Fällen sollten die bildgebenden mit den klinischen Befunden und Lungenfunktionstests korreliert werden. Bei Patienten, bei denen sich die residualen Veränderungen im Thorax-CT nicht auflösen, ist eine wiederholte Nachuntersuchung nach weiteren 3 Monaten ratsam. Es wird empfohlen, Patienten mit persistierenden Symptomen nach COVID-19 an spezialisierte multidisziplinäre Post-COVID-19-Kliniken oder -Ambulanzen anzubinden und eine zielgerichtete Rehabilitation zu ermöglichen.

## Fazit für die Praxis

- Bei den Patienten nach COVID-19 können pulmonale Symptome, Lungenfunktionsbeeinträchtigungen und radiologische Veränderungen auftreten.
- Nach Abklingen der akuten Phase einer COVID-19-Pneumonie kommt es 3 bis 4 Wochen nach der Entlassung bei mehr als der Hälfte der Patienten zu einer vollständigen Rückbildung der Lungenparenchymveränderungen.
- In der CT sind die Milchglasareale und die strangförmigen Verdichtungen die häufigsten residualen Veränderungen nach einer COVID-19-Pneumonie, die histologisch einer organisierenden Pneumonie entsprechen. Diese Veränderungen sind ausgeprägter nach einer schweren Erkrankung und bei der Mehrheit der Patienten innerhalb von 12 Monaten nach Genesung regredient.
- Ein Teil der Patienten nach schwerer COVID-19-Pneumonie kann im Verlauf *fibroseähnliche* Veränderungen entwickeln, die sich jedoch mit hoher Wahrscheinlichkeit im Laufe der Zeit wieder zurückbilden.
